# Whole-genome sequencing and determination of the properties of 18 strains of the genus *Bacillus* isolated from permafrost in Yakutia

**DOI:** 10.1128/mra.00751-25

**Published:** 2025-10-22

**Authors:** Yulia Goncharova, Kseniya Khlopova, Vera Evseeva, Irina Bakhteeva, Raisa Mironova, Yuriy Skryabin, Angelina Kislichkina, Angelika Sizova, Ivan Dyatlov, Vitalii Timofeev

**Affiliations:** 1 Laboratory of Anthrax Microbiology, State Research Center for Applied Microbiology and Biotechnology (SRCAMB), Obolensk, Russia; 2 Department of Molecular Microbiology, State Research Center for Applied Microbiology and Biotechnology (SRCAMB), Obolensk, Russia; 3 Culture Collection Department, State Research Center for Applied Microbiology and Biotechnology (SRCAMB), Obolensk, Russia; 4 State Research Center for Applied Microbiology and Biotechnology (SRCAMB), Obolensk, Russia; Fluxus Inc., Sunnyvale, California, USA

**Keywords:** permafrost, *Bacillus cereus*, draft genome, MLST, WGS

## Abstract

Here, we describe 18 strains of bacilli isolated from permafrost. Most strains belong to non-anthracis species of the *Bacillus cereus* complex. Some strains exhibit atypical properties such as sensitivity to anthrax bacteriophages and lack of hemolytic activity and have unique MLST sequence types.

## ANNOUNCEMENT

*Bacillus cereus* complex includes several closely related species of the genus *Bacillus* (1), which can cause a wide range of diseases, from food poisoning to anthrax (2). Most studies have focused on anthrax and its causative agent, *B. anthracis*. Other species remain poorly studied. We describe 18 *Bacillus* strains isolated from a soil sample taken from permafrost in the Sakha Republic (Yakutia), Russian Federation on the bank of the Uyandina River (68.564567°N 144.769827°E). Permafrost soil samples were obtained by the Laboratory of Especially Dangerous Infections of Center for Hygiene and Epidemiology in Yakutia and sent to SRCAMB without temperature control in December 2016. We isolated three *B. anthracis* strains from it (3). The remainder of the soil was frozen at −70°C until re-examination. Additionally, we isolated 18 strains of bacilli and *B. anthracis* strain in 2021–2024 (4). Cultures were isolated by seeding soil samples, preliminarily thawed at room temperature and heated by boiling, onto BHI-agar (Obolensk, Russia) and incubated at 20°C for 48 h. The isolated strains were preserved by freezing spore cultures in 15% glycerin at −70°C.

A typical non-anthracis strain of *B. cereus* complex is motile, resistant to anthrax bacteriophages, and has lecithinase, phosphatase, and hemolytic activities. Atypical properties, more typical of *B. anthracis*, were found for several strains (Table 1, https://doi.org/10.6084/m9.figshare.30136108.v1).

**TABLE 1:** Characteristic of the strains isolated from permafrost in Yakutia and sequencing^*a*^ statistics

Strain	Motility	Lecithinase activity	Phosphatase activity	Hemolytic activity	Strong odor during growth	Sensitivity to anthrax phage gamma A-26	Sensitivity to anthrax phage B-Fah	SRA accession no. (raw data)	No. of reads	No. of bases	GenBank accession no.	GenBank assembly	Total length (bp)	Genome coverage	No. of contigs	*N*_50_	G+C content (%)	No. of proteins	MLST-ST	panC-group
*B. cereus* YakM1	+	+	+++	+++	-	-	-	SRR33640642	15,902,495	6,119,052,118	JAKGBS02	GCA_022458945.2	5,686,025	1,124	70	254,664	34.84	5,532	857	IV
*B. mycoides* YakM1-1	+	+	+++	+++	-	-	-	SRR33930845	6,759,514	2,021,493,406	JBFNPR02	GCA_040797105.2	6,201,140	351	129	354,990	35.12	6,036	3,357	VI
*B. mycoides* YakM2	+	+	+++	+++	+	-	-	SRR33640641	2,680,559	1,021,772,223	JAKGBR02	GCA_022458665.2	5,985,964	189	94	483,631	35.20	5,797	3,357	VI
*B. wiedmannii* YakM3	+	+	+++	+++	+	-	-	SRR33640640	9,173,172	3,513,218,436	JAPJTU02	GCA_026275445.2	5,867,123	649	93	260,973	35.05	5,764	217	II
*B. mycoides* YakM4	+	+	+++	-	-	-	-	SRR29660467	2,380,873	695,673,068	JBFNPQ02	GCA_040797065.2	6,199,737	112	128	409,395	35.12	6,028	3,357	VI
*B. wiedmannii* YakM7	+	+	+++	+++	-	+	-	SRR33640639	1,189,096	349,109,772	JAKGBQ01	GCA_022458675.1	5,845,085	71	66	257,594	35.05	5,716	217	II
*B. pacificus* YakM8	+	+	+++	+++	-	-	-	SRR33640638	13,446,660	5,100,260,172	JAPJTT02	GCA_026275465.2	5,480,132	958	69	296,945	35.25	5,477	32	III
*B. pacificus* YakM9	+	+	+++	+++	-	-	-	SRR33640637	9,946,569	3,139,609,769	JAPJTS02	GCA_026275395.2	5,477,489	930	57	296,945	35.24	5,471	32	III
*B. wiedmannii* YakM10	+	+	+++	+++	-	+	-	SRR33640636	3,008,983	927,507,095	JAKGBP02	GCA_022458685.2	5,860,071	288	98	260,978	35.04	5,762	217	II
*B. cereus* YakM11-1	+	+	-	-	-	-	-	SRR29660465	4,133,506	1,203,594,412	JBFNPO01	GCA_040797085.1	5,811,491	207	96	260,979	35.04	5,701	217	II
*B. cereus* YakM11-2	+	+++	+	+	-	-	-	SRR29660464	1,334,835	380,863,435	JBFNPN01	GCA_040796985.1	5,466,011	70	111	133,829	35.26	5,471	32	III
*B. paranthracis* YakМ13	+	+	+++	+++	-	-	-	SRR33640635	5,889,510	2,209,808,655	JAPJTR02	GCA_026275425.2	5,256,083	427	60	462,371	35.32	5,275	3,358	III
*B. wiedmannii* YakМ14	+	+	+++	+++	-	+	-	SRR29660462	23,946,430	8,888,979,737	JBFNPL01	GCA_040797035.1	5,860,001	1,516	115	181,908	35.17	5,784	217	II
*B. wiedmannii* YakМ15	+	+	+++	+++	-	+	-	SRR33640634	2,939,867	1,100,946,363	JAKGBO01	GCA_022458695.1	5,833,972	207	70	209,776	35.03	5,715	217	II
*B. atrophaeus* YakМ16	+	-	-	+++	+	-	-	SRR33371954	13,095,330	3,886,836,159	JBNLXU01	GCA_050171665.1	4,303,474	158	37	477,792	43.00	4,141	-	-
*B. atrophaeus* YakМ17	+	-	-	+++	-	-	-	SRR33371953	3,906,599	1,162,451,074	JBNLXT01	GCA_050171645.1	4,313,758	206	46	440,326	42.99	4,168	-	-
*B. atrophaeus* YakМ18	+	-	-	+++	+	-	-	SRR33371952	17,207,500	5,119,084,788	JBNLXS01	GCA_050171565.1	4,319,695	183	44	440,326	42.99	4,165	-	-
*B. wiedmannii* YakМ22	+	+	+++	+++	-	-	-	SRR33371951	1,582,345	470,750,182	JBNLXR01	GCA_050171545.1	5,798,561	84	177	135,937	35.09	5,714	217	II

^*a*^ -, absent; +, present; +++, present, feature is strongly expressed.

Single colonies were isolated on BHI-agar (Obolensk, Russia) at a temperature of 20°C for 24 h. One colony was picked for DNA extraction using the Genomic DNA Purification Kit (Thermo Fisher Scientific, USA) following the manufacturer’s instructions. DNA was quantified using a NanoDrop One spectrophotometer (Thermo Fisher Scientific), and its quality was assessed by electrophoresis in 0.8% agarose gel.

The MGIEasy FS DNA Library Prep Kit was used to prepare DNA libraries. Genome sequencing was carried out using the DNBSEQ-G400 platform (MGI Tech Co., Ltd, China) with 2000RS High-throughput Sequencing Kit PE200 (MGI Tech Co., Ltd) following the manufacturer’s protocols. All generated reads were *de novo* assembled using Unicycler v.0.4.7 with default settings (5). Unicycler applies SPAdes' built-in error correction to short reads, which can adjust or discard low-quality sequences. No additional external trimming or quality filtering was performed prior to assembly. Genomes were annotated using the NCBI Prokaryotic Genome Annotation Pipeline v.6.7 (6). According to species identification during the procedure of depositing into NCBI Genome, three strains were identified as *B. atrophaeus*, and the remaining 15 strains belonged to the *B. cereus* complex (Table 1) (7).

MLST sequence types (MLST-ST) of *B. cereus* complex strains were determined using the PubMLST database service (8). The strains belong to five MLST-STs, and two of them were identified for the first time. MLST-ST3357 represents a combination of previously known gene alleles. MLST-ST3358 contains newly identified alleles of genes glp453 and ilv501. New alleles of loci and MLST-STs were registered in PubMLST. As an important phylogenetic and taxonomic characteristic, panC group of the strains was defined (Table 1) (9, 10).

## Data Availability

The genome sequences and sequence reads were deposited in the GenBank database under the accession numbers listed in Table 1.
